# Influence of Habitat Types on Diversity and Species Composition of Urban Flora—A Case Study in Serbia

**DOI:** 10.3390/plants10122572

**Published:** 2021-11-25

**Authors:** Milan Glišić, Ksenija Jakovljević, Dmitar Lakušić, Jasmina Šinžar-Sekulić, Snežana Vukojičić, Milena Tabašević, Slobodan Jovanović

**Affiliations:** 1 Academy of Applied Studies Šabac, Dobropoljska 5, 15000 Šabac, Serbia; 2 Faculty of Biology, Institute of Botany and Botanical Garden, University of Belgrade, Takovska 43, 11000 Belgrade, Serbia; kjakovljevic@bio.bg.ac.rs (K.J.); dlakusic@bio.bg.ac.rs (D.L.); jsekulic@bio.bg.ac.rs (J.Š.-S.); sneza@bio.bg.ac.rs (S.V.); milenatabasevic@gmail.com (M.T.); sjov@bio.bg.ac.rs (S.J.)

**Keywords:** species composition, richness, urban areas, anthropogenic impact, climate

## Abstract

The aim of this study was to investigate the floristic composition and diversity of seven urban habitat types in 24 Serbian cities with different climatic affiliation. In each of the 24 cities, we selected 1 ha plots representing a habitat from one of the following groups: square, boulevard, residential area with compact and with open building pattern, city park, and sites with early and mid-succession vegetation stages. All vascular plant species that occur spontaneously in these plots were observed. Data on the main climatic characteristics were collected for each plot, and data on the life forms were obtained for each species recorded. Diagnostic species were identified for each habitat type analyzed, and alpha, beta and gamma diversity were calculated. A total of 674 taxa were recorded in the studied area. Significant differences were observed in habitats by diagnostic species and by life form representation. The lowest alpha and gamma diversity and the dominance of therophytes were observed in habitat types with intensive anthropogenic impact, whereas the highest number was recorded in mid-successional sites and residential areas with a compact building pattern. The analysis showed that habitat type influences species composition much more than climate.

## 1. Introduction

Human activities are an inseparable part of urban area and play a leading role in modifying its ecological characteristics, forming similar conditions in diverse, often remote areas. Hence, the similar urban habitats are found in the vast majority of cities, even in areas in different biogeographical regions, with different macroclimatic characteristics [1,2]. Large-scale introduction of species with the cosmopolitan type of distribution, sometimes associated with a decline in native species, may lead to a further increasing similarity in species composition between regions [3,4]. Additional homogenization is caused by the presence of invasive species, primarily archeophytes, whereas neophytes mainly lead to opposite effects [5,6]. However, both groups of invasive species have been shown to contribute to an increase in the richness of plant species in urban habitats [5,7]. Namely, according to Pyšek [8], archaeophytes and neophytes account for 15% and 25%, respectively, of the urban flora in Central Europe, although the negative effects of alien species on native diversity have also been observed [9]. Additionally, urban areas are very heterogeneous, so this also contributes to a larger number of species in cities [10,11]. This heterogeneity, caused by different disturbance regimes, induced differences in species composition [12]. Simultaneously with the certain similarities and the number of generalist species found both in and outside the cities, pronounced differences can be observed compared to the surroundings, wherefore the cities can be regarded as a kind of ecological island [6].

There are many characteristics of urban habitats that distinguish them from natural ones: higher levels of disturbance [13,14], herbicide use [15], air and soil pollution [16,17], nutrient enrichment [5,18], higher temperatures due to the urban heat island effect [19,20], higher input of alien species propagules [21], etc.

There are a number of reasons why urban flora research is attracting increasing attention: most of Europe’s human population lives in urban areas [22]; the plants of urban habitats contribute to ecosystem services and affect the citizens’ well-being [15,23]; urban areas can be centers for the spread of allochthonous plant species to neighboring territories [8,24]; and urban habitats can serve as a refugia for plant species, even those considered rare or endangered [11]. The number of studies dealing with urban flora has increased considerably in recent decades [25]. Comparative studies of urban flora in large areas and the implementation of standardized sampling protocols made a great contribution to the understanding of the distribution and ecology of plants in central European cities [5,6,12,26,27,28,29], indicating striking differences between urban habitats in terms of plant species diversity, induced by the types of urban habitats, climate, and specific spatial patterns [26,30,31]. However, the results from Central Europe are hard to generalize to the whole of Europe. For example, diversity of plant species in urban habitats in Southern Europe is greater compared to the parts of Central Europe with a different climate [30]. In addition, the urban flora of Southeastern Europe is poorly studied compared to other parts of Europe, with previous studies often focusing on individual cities or specific urban habitats within them [25]. Comparative and comprehensive studies of urban flora in SE Europe are particularly rare and are not based on standardized sampling methods, rather on a comparison of existing data [32]. For the above reasons, large-scale comparisons and generalizations of features and trends in the urban flora of Southeastern Europe are still lacking.

The aim of this study was therefore to investigate the floristic composition and diversity of urban habitats in Serbia using the standardized protocol established by Lososová et al. [26], to obtain comparable results. Cities in Serbia are a good model for such a study, as climatic differences between cities are considerable. Hence, our aim was also to investigate (1) how urban habitat types affect floristic composition and diversity and (2) which factor has a stronger effect on floristic composition, habitat types, or climatic characteristics.

## 2. Results

Significant differences in species composition were observed among selected habitat types. Diagnostic species for each habitat type are listed in Table 1. The highest average value of Φ (0.13) and the highest number of diagnostic species (67) were calculated for mid-successional sites (m). This habitat type was dominated by deciduous shrubs and trees (e.g., *Prunus spinosa*, *Cornus sanguinea*, and *Juglans regia*), but also by a large number of herbaceous perennial species (e.g., *Hypericum perforatum*, *Dipsacus laciniatus*, *Rumex patientia*, *Agrimonia eupatoria*) and grasses (e.g., *Calamagrostis epigejos*, *Poa trivialis*, *Holcus lanatus*, *Bromus hordeaceus*). Early successional sites and residential areas with compact building patterns were also characterized by a relatively high average value of Φ (0.10, both) and a relatively high number of diagnostic species (39, both). The diagnostic species with the highest Φ value (>0.50) in early successional sites were *Papaver rhoeas*, *Bromus hordeaceus*, *Polygonum lapathifolium*, and *Vicia cracca*. Residential areas with compact building patterns were mostly characterized by ornamental species, which spread from neighboring gardens (e.g., *Campsis radicans*, *Kerria japonica*, *Antirrhinum majus*, *Ipomoea purpurea*, *Rudbeckia hirta*). In residential areas with an open building pattern, 22 diagnostic species were observed, with domination of ornamental species (e.g., *Mirabilis jalapa*, *Commelina communis*, *Hibiscus syriacus*) and an average Φ value of 0.7. Only five diagnostic species were determined for city parks (p) and the species with the highest Φ value was *Quercus robur*, which often occurs as seedlings. Boulevards (b) hosted a large group of different plants, but only four species were determined as a diagnostic. By comparison, no positive correlation with city squares (s) was found for any of the plant species, and the lowest average value of Φ was obtained (0.01) for this habitat type.

**Figure 1:**
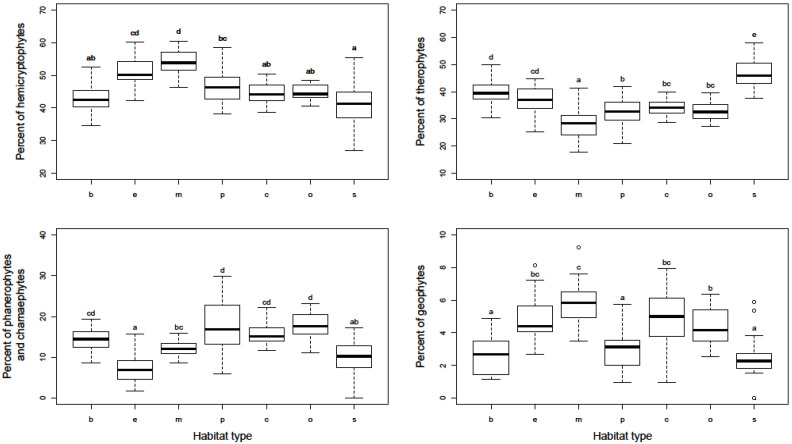
Proportion of hemicryptophytes, therophytes, phanerophytes/chamaephytes, and geophytes in particular urban habitat types. X-axis abbreviations: b—boulevard, e—early successional sites, m—mid-successional sites, p—city park, c—residential area (compact building pattern), o—residential area (open building pattern), s—historical city square. Homogeneous groups of urban habitat types are denoted by the same letters (*p* < 0.01).

The habitat types also differed significantly in the representation of plant life forms (Figure 1). The largest participation of hemicryptophytes, as most represented compared to the other life forms, was found in early and mid-successional sites, and the lowest at squares and boulevards. By comparison, therophytes had the largest share in squares and the lowest in mid-successional sites. Parks and residential areas, both compact and open, were characterized by a somewhat higher proportion of phanerophytes and chamaephytes compared to other habitat types. Geophytes were generally the least represented, especially in mid-successional sites.

**Figure 2:**
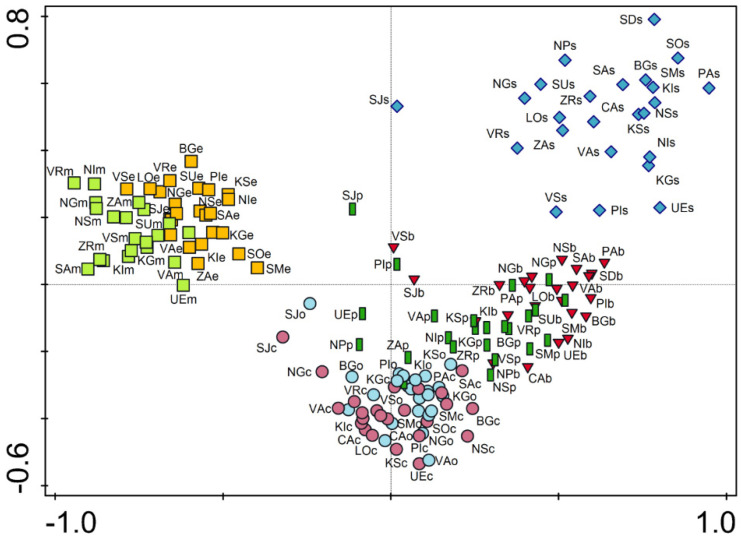
PCA ordination of plots according to plant species composition. Eigenvalues: axis 1, 0.2492; axis 2, 0.0925. Abbreviations: the first two uppercase letters represent the city code (see Table 2), the third lowercase letter represents the habitat type (see Material and Methods). Legend: red triangles—boulevards, orange squares—early successional sites, light green squares—mid-successional sites, dark green boxes—parks, purple circles—residential area (compact building pattern), light blue circles—residential area (open building pattern), blue diamonds—squares.

The ordination diagram obtained by PCA indicated a predominant grouping of plots belonging to the same habitat type, with a certain overlapping between some of them (Figure 2). The city square (s) distinguished most clearly from the others, with no overlap of this habitat type with any other habitat type analyzed. The most distant from the other habitat types were the plots representing mid-successional and early successional sites. These two habitat types formed a well-separated group, with minor overlapping between each other. The differences were much less noticeable between other habitat types, particularly between city parks (p) and boulevards (b) and between the two types of residential areas (c and o).

The results of the RDA analysis indicated that species composition was affected by both habitat type and climatic variables, with the greater influence of habitat type (9.7%) in relation to climatic characteristics (2.3%), whereas shared variance was not observed (Table 3).

The total number of taxa recorded on 164 plots in the 24 investigated cities on the territory of Serbia was 674, with an average of 105 taxa observed per plot. The highest number of taxa was recorded in the residential areas with compact building pattern (c) in Niš (147), Kruševac (146), and Loznica (142), whereas the lowest number of registered taxa was observed in the city squares (s) of Smederevo and Pančevo (38) and Sremska Mitrovica (42).

**Figure 3:**
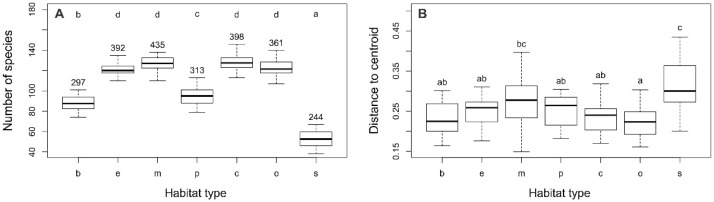
(**A**) Alpha (box plots) and gamma (numbers above) diversity in particular urban habitat types. (**B**) Beta diversity of plant taxa within urban habitat types. X-axis abbreviations: b—boulevard, e—early successional sites, m—mid-successional sites, p—city park, c—residential area (compact building pattern), o—residential area (open building pattern), s—historical city square. Homogeneous groups of urban habitat types are denoted by the same letters (*p* < 0.01).

There were considerable differences in alpha, beta, and gamma diversity between the habitat types studied. The lowest alpha diversity was found in the city squares (s; 53 plant taxa) and boulevards (b; 88 taxa), whereas the highest values were recorded in residential areas with compact building patterns (c; 128 plant taxa) and at the mid-successional sites (m; 127 plant taxa). Similarly, the lowest gamma diversity was found in the city squares (s; 244 plant taxa) and boulevards (b; 297 taxa), whereas the highest values were found at the mid-successional sites (m; 435 plant taxa) and residential areas with compact building pattern (e; 398; Figure 3A). The highest beta diversity was recorded within city squares (s) and the lowest in residential areas with open building patterns (c; Figure 3B).

A strong positive statistically significant correlation was found between alpha and gamma diversity (r = 0.93, *p* = 0.002). Alpha and beta diversity were strongly negatively and statistically significantly correlated (r = −0.82, *p* = 0.02). Gamma and beta diversity were also negatively correlated, but this correlation was statistically insignificant (r = −0.62, *p* > 0.1).

## 3. Discussion

Significant differences in species composition were found between individual types of urban habitats. As observed from the results of ordination analysis and the plots grouping, species composition is primarily influenced by urban habitat type and intensity of human influence, much more than by climatic features. This is consistent with the findings of Lososová et al. [26], who analyzed the urban flora of Central European cities along a gradient of distinctly different biogeographical regions with contrasting climatic characteristics, and with the results of Rebele [1] and Savard et al. [2], which confirm the hypothesis of uniformity of the urban environment.

Based on the ordination analysis of species composition, plots were grouped primarily based on habitat type affiliation. Four groups of plots can be observed: plots representing squares (1), plots representing boulevards and parks (2), plots representing residential areas (3), and plots representing successional sites on the urban peripheries (4). This suggests that certain habitat types have greater similarities in species composition than others. These similarities are the result of a similar character of anthropogenic influence and the location of the plots in the city (center or periphery; Figure 2).

The results of alpha and gamma diversity analysis in the urban habitats of the studied area showed that the lowest number of species was found in city square, which is consistent with the results from Central European cities [6,27]. Sealed and paved surfaces, which dominate in city squares are hostile habitats, and a relatively small number of plant species are adapted to such extreme conditions (trampling, high insolation, drought, etc.). Hence, it is not surprising that therophytes, as a disturbance tolerant life form, have the largest share in city squares (Figure 1). However, although well-adapted to highly influenced urban habitats, therophytes are thought to be more prone to extinction compared to other life forms [33,34]. Intensive human influence and vegetation limited to small patches strongly support the presence of cosmopolitan and even alien species, which negatively affect the native flora and consequently leads to their local extinction [34,35]. The richness of native flora is additionally affected by environmental filters that have to be overcome in order for plants to arrive from the surrounding natural habitats [12]. Furthermore, these habitat types have a significant share of alien and ornamental plants that have been spread by human activities. Due to all this, squares are unique in their species composition compared to the other urban habitat types, as indicated by their clear separation from them in the ordination diagram (Figure 2).

As opposed to a small number of species in city squares in Serbia and in Central European cities [27], the high plant species richness has been observed in the old centers of the Italian Mediterranean cities [30], due to a number of plants growing undisturbed on the ancient walls. However, such microhabitats beyond intense anthropogenic influences are rare in the city squares in Serbia, and chasmophytic flora were represented by only a few species (e.g., *Cymbalaria muralis* and *Sedum* spp.). Simultaneously with the lowest alpha and gamma diversity, urban squares in Serbia had the highest beta diversity, indicating that the differences between specific habitats of this type were greater than in other habitat types. The same was found in Central European cities as a result of a very low number of species [26].

A somewhat higher floristic richness was observed in boulevards. Bearing in mind that boulevards are usually spatially connected to city squares, the human influence is similar to that for squares but of lower intensity, which resulted in somewhat higher species richness. A major contributor to species richness in boulevards is tree lines, especially those with unsealed surfaces around the trees, considering that these microhabitats harbored a wide range of plant species. Due to the presence of planted trees, a significant number of their seedlings were observed in boulevards. This is one of the reasons for the marked similarities in the species composition of boulevards and parks, whose plots are grouped in the ordination diagram (Figure 2). The species detected in this habitat type are generally common in the cities and almost half of them were detected in all the habitat types analyzed, whereas only nine were recorded only in the boulevards, confirming that the species found in the boulevards are those that generally survive very successfully in urban conditions [12]. Similar to the city squares, the boulevard’s flora were characterized by a significant participation of therophytes, particularly compared to the other habitat types. However, in addition to therophytes, boulevards were also characterized by perennials adapted to growing in small cracks in concrete (e.g., *Sagina procumbens*) and seedlings of tree species, primarily those deliberately planted (e.g., *Acer pseudoplatanus*). In contrast, in city squares and boulevards, the least represented life form was geophytes (Figure 1). Although bulbs and rhizomes enable them to survive in the hostile environment [36], their numbers were found to be negatively correlated with the number of inhabitants and traffic density [32].

Parks are very specific urban habitats. Although they resemble natural forest habitats (e.g., similar mild-mesoclimate, similar light regime, etc.), with a number of typical forest plants that can be found in them (e.g., *Brachypodium sylvaticum*, *Clematis vitalba*, seedlings of *Quercus robur*, etc.), parks are artificial habitats created by deliberate planting of trees, often of non-native origin and heavily impacted by human activity (e.g., trampling, regular mowing, planting horticultural annuals and perennials, etc.). In addition to the planted tree species, city parks in Serbia are also characterized by significant participation of spontaneously growing ornamental alien plants, especially typical ruderal ones (e.g., *Amaranthus deflexus* and *Lactuca serriola*, etc.), but also shrubs and tree seedlings (e.g., *Philadelphus coronarius*, *Symphoricarpos albus*, and *Broussonetia papyrifera*). Hence, in addition to phanerophytes as the predominant life form, a significant number of hemicryptophytic species occur in this habitat type (Figure 1). Contrary to almost half of the species detected in the urban parks that were found in all seven groups of the analyzed urban habitat types, a total of 19 species, mainly tree and shrub species, were detected exclusively in this habitat type. However, alpha and gamma diversity in city parks in Serbia was not high, especially compared to residential areas and early and mid-successional sites, primarily because their homogeneity and the lack of specific microhabitats that could harbor different species types. Regardless of this, city parks are recognized as very important habitats for conservation of local biodiversity [37,38,39].

Residential areas of cities in Serbia, especially those with compact building patterns, harbor a wide range of species, with the highest alpha and significant gamma diversity observed in this type of urban habitat. This high diversity is a consequence of the marked heterogeneity of habitats, considering that residential areas with densely distributed individual housing units represent a mosaic of diverse habitat types: sidewalks and other paved and sealed surfaces, lawns with different mowing regimes, ornamental gardens, urban gardens with various cultivated plants and accompanying weed flora, tree lines, etc., which contribute significantly to the high species richness, especially when compared to squares, boulevards, and urban parks [40]. However, it should be considered that the shape of the sampling plot may also have an influence on increasing species richness and that elongated plots, such as those used to study of this type of habitat, tend to have more species than compact plots of the same size [41,42,43]. Although our analyses revealed that the highest alpha diversity was found in residential areas with compact building patterns, Godefroid and Koedam [44] indicated a contrasting pattern, i.e., that half open and open areas in Brussels promote species richness, whereas areas with compact structures lead to its reduction. Due to the complexity of these habitat types, residential areas in analyzed cities in Serbia were characterized by different groups of plants, including species commonly found in squares or boulevards (e.g., *Arenaria serpyllifolia* and *Eragrostis minor*), a wide range of grasses (e.g., *Poa* spp. and *Lolium perenne*), escaped ornamental plants (e.g., *Kerria japonica* and *Antirrhinum majus*), juveniles of crop plants (e.g., *Zea mays* and *Solanum lycopersicum*), crop weeds (e.g., *Cynodon dactylon* and *Elymus repens*), and spontaneously growing native and alien trees and shrubs (e.g., *Campsis radicans*, *Syringa vulgaris*, and *Rhus typhina*).

Urban habitat types that stood out in terms of species composition and plant diversity were successional sites, particularly mid-successional ones, abandoned long enough to form species-rich grasslands. Similar findings have been made in several other urban studies [26,44]. Considering that these habitats usually develop on the urban periphery, they are characterized by the absence of strong human impact, with the main difference between them being the duration of the disturbance-free period. Due to recent disturbance, early successional sites are characterized by a vegetation cover dominated by annual plants with ruderal life strategies, capable of rapid colonization of bare ground (e.g., *Bromus tectorum* and *Petrorhagia prolifera*). Additionally, the results of previous studies [35,45] show that compared to the older succession stages, the younger ones are more susceptible to alien species invasion. Mid-successional sites hosted a higher number of plant species than early successional ones and are characterized by the highest alpha diversity compared to the urban habitat types studied. Because of a longer period of non-disturbance, these sites are suitable for inhabiting species with different life strategies. Hence, a lower proportion of therophytes was observed at the mid-successional sites compared to the early successional ones, whereas both habitat types were characterized by a greater participation of hemicryptophytes and therophytes in relation to the other types analyzed (Figure 1). Additionally, proximity to natural vegetation and openness to the urban surroundings facilitated the influx of native species into these habitats, and various shrub and tree species are common in these habitat types (e.g., *Prunus* spp., *Ulmus* spp., and *Juglans regia*). In general, mid-successional sites are characterized by a higher share of native flora than other urban habitat types, particularly city squares and boulevards [5,35]. Despite the very different physiognomy, early and mid-successional sites have a very similar species composition. Many of the same species of shrubs and trees are found in both types of successional sites, but in the early successional sites they appear as seedlings and much smaller individuals. Additionally, their similarity is also contributed by the fact that due to the complex human influence of varying intensity, some patches of older vegetation were found in the early successional sites analyzed because it was hard to find a completely uniform 1 ha area.

Although the results of this study indicated that local site conditions are the predominant factors determining plant species composition, the results of similar studies of urban flora in Italy contradict this hypothesis because climate was found to be the main factor determining species composition, most likely due to the strong climatic gradient from the north to the south of the Apennine Peninsula [30]. Despite the fact that the climate in Serbia is very diverse, ranging from continental to sub-Mediterranean and mountainous, the differences are not pronounced enough to point to climate as the main factor determining the diversification of flora in urban habitats, which is also true for the Central European urban flora [26]. However, the effect of the climate on the composition of plant species in urban habitats in Serbia should not be neglected. This applies particularly to Sjenica, which is located at an altitude of 1026 m and is characterized by a humid mountain alpine climate (Table 2), as the ordination analysis indicated a grouping of several plots of this city. The climatic differences between the other cities analyzed are less noticeable, resulting in a greater similarity in the ordination diagram (Figure 2). Apart from the climate, the composition of species in urban habitats may be influenced by other factors, such as the geographical location, and the structure of the city and its historical features. However, as their effects can be significant, especially in large-scale studies [27], and almost negligible in studies focusing on cities from a smaller geographical area, these factors are usually neglected in the analyses.

## 4. Materials and Methods

### 4.1. Data Sampling

Investigation of urban flora was carried out in 24 cities in Serbia (Table 2). Cities were selected to represent all major climate types and subtypes in Serbia. An additional condition that cities had to meet was the existence of the preselected typical urban habitat types. According to Stevanović and Stevanović [46], the following climate types and subtypes are represented in the territory of Serbia (climate type and subtype designations, according to Walter and Leith [47] and Horvat et al. [48], are given in parenthesis): transitional submediterranean-Aegean subcontinental (IV6), semi-arid continental Pannonian (VII), semi-humid continental Danubian (VII), transitional subcontinental-semiarid continental (VI3b/VII), semi-arid temperate continental (subcontinental)—central-southeastern Balkan or Moesian (IV3), humid temperate-continental—west Balkan or Illyrian (IV2b), and humid mountain alpine (XI).

To make comparable samples, floristic data were collected using a standardized protocol, which has already been used in similar studies for Central European cities [5,6,12,26,27,28,29]. In each of 24 selected cities in Serbia, we recorded the plant composition at seven specific sites of 1 ha in size. Each of the seven sites represents a different type of urban habitat:

1. historical city square (s), mostly with the buildings constructed before 19th century; sealed or paved more than 90% of the total area.
2. boulevard (b), with the buildings from 19th century, tree lines and small-size lawns; sealed or paved more than 70% of the total area.
3. residential area, with compact building pattern (c), represented by family houses (at least 50 years old) and private yards.
4. residential area, with open building pattern (o), represented by apartment blocks (40-60 years old), and lawns with sparse shrubs and trees.
5. city park (p), with coverage of old deciduous trees from 10 to 50% and regularly mowed lawns.
6. early successional sites (e), severely disturbed in the last 1–3 years, dominated by bare soil and scarce vegetation cover.
7. mid-successional sites (m), abandoned 5 to 15 years ago, with predominance of perennial grassland, and sparse young trees and shrubs.

Adequate sites (habitats) were selected using maps and satellite images in Google Earth. The 1 ha plots were selected within each habitat type and all vascular plants were recorded within them. This included seedlings from spontaneously grown planted trees and garden plants. However, intentionally planted individuals were omitted. In residential areas with a compact building pattern (c), a different approach was applied due to limited access to private gardens. In these cases, instead of an area of 1 ha, transects along a street 500 m long were analyzed, recording all species found in accessible areas, in addition to all those that could be seen from the street inside private yards. The research was conducted in 2015–2019 in the period from June to the end of August, to avoid spring and autumn, i.e., plants with significant variations in phenology. The nomenclature of species corresponding to the diagnostic species of classes of plant communities dominated by vascular plants follows Electronic Appendix S6 (EVC1) of Vegetation of Europe [49], and for other species follows the nomenclature of Flora Europaea [50].

### 4.2. Data Analysis

The composition of plant species recorded within individual habitat types was shown in synoptic tables. To determine diagnostic species for particular habitat types, the phi coefficient of association (Φ) was used as a statistical measure of the concentration of occurrence of species in particular habitat types [51]. Diagnostic species for a particular habitat type were defined as species that preferentially occur in that habitat type. Fisher’s exact test (*p* < 0.05) was used to assess the statistical significance of the species-habitat association, quantified by Φ, as shown in the following equation [52]:

$$\Phi =\frac{N\times n_{p}-n\times N_{p}}{\sqrt{n\times N_{p}\times \left(N-n\right)\times \left(N-N_{p}\right)}}$$

where N is the number of all sites in the data set, N_p_ is the number of sites in the particular habitat type, n is the number of occurrences of the species in the data set, and n_p_ is the number of occurrences of the species in the particular habitat type. Diagnostic species were considered to be those that had a statistically significant species–habitat association and Φ > 0.30. Synoptic tables and calculations of phi coefficient were carried out in the JUICE program [53].

All recorded species were categorized into four different groups according to their life form: geophytes, phanerophytes and chamaephytes (trees and shrubs), therophytes, and hemicryptophytes [54]. To compare the differences in the frequency of individual life forms in relation to urban habitat types, ANOVA [55] was used.

According to detrended correspondence analysis (DCA), the length of the gradient in species composition was 2.27 SD units. Therefore, to assess the general variation patterns in composition of plant species among urban habitat types, unconstrained and constrained linear ordination methods were used (PCA and RDA, respectively). Analysis and visualization of the PCA and RDA diagrams were performed using CANOCO 5.12 program [56].

To distinguish the influences of climatic variables and urban habitat types on plant composition, redundancy variation partitioning for RDA was carried out [57]. Two groups of variables were collected for each site: (1) habitat type, which was given as a categorical variable with seven expressions, and (2) three climatic variables: mean annual temperature, annual temperature range, i.e., the difference between mean temperatures in July and January, and annual precipitation total. The climate variables were taken from the WorldClim dataset. The significance of the influence of climatic variables and urban habitat types was tested by Monte Carlo permutations (999 permutations). These calculations were performed in the CANOCO 5.12 program [56].

Alpha and gamma diversity were used to indicate differences in plant species richness between habitat types. Alpha diversity was defined as the average number of taxa recorded per plot in each habitat type, and gamma diversity was estimated as the total number of taxa observed in all plots belonging to a particular habitat type. To determine beta diversity, we calculated an index of beta diversity: S/a-1, where S is the total number of taxa, and a is the average number of taxa per plot. The calculations of alpha, gamma, and beta diversity and the visualization of their differences between habitat types were carried out in the “vegan” package of the R programming language [58].

## 5. Conclusions

The results of the study of urban flora of Serbia indicate significant floristic richness of the investigated areas. Pronounced differences were found between the analyzed habitat types, both in terms of diversity, species composition, and dominant life forms. It was shown that the species composition and dominance of particular life forms are directly related to the intensity of anthropogenic influence. The lowest alpha and gamma diversity was found in city squares and boulevards, with a dominance of therophytes, a life form related to disturbed habitats, whereas habitats in the urban periphery under less pronounced human influence and heterogeneous residential urban areas, are characterized by significant floristic richness and much more uniform distribution of life forms. Comparison of the influence of different factors on the composition of urban flora shows that urban flora in the analyzed cities is more influenced by the type of habitat than by climatic features. Our results indicated that the most important ways to increase plant diversity in cities are the following: (1) allowing natural succession and reducing the intensity of the anthropogenic factor in certain parts of the city; (2) providing greater heterogeneity of urban areas, and forming specific urban microhabitats that allow the survival of those species that are not typical urbanophiles.

## Figures and Tables

**Table 1:** Diagnostic plant species for studied urban habitat types in Serbia.

Habitat Type	Taxon
Boulevard	*Sedum lineare* (0.44), *Sagina procumbens* (0.32), *Chelidonium majus* (0.31), *Acer pseudoplatanus* (0.30)
Early successional site	***Papaver rhoeas* (0.54), *Bromus hordeaceus* (0.53), *Polygonum lapathifolium* (0.51), *Vicia cracca* (0.50)**, *Onopordum acanthium* (0.49), *Tanacetum vulgare* (0.49), *Tragopogon dubius* (0.47), *Sisymbrium loeselii* (0.47), *Rumex crispus* (0.45), *Linaria vulgaris* (0.45), *Phragmites australis* (0.44), *Petrorhagia prolifera* (0.42), *Silene vulgaris* (0.41), *Rumex patientia* (0.41), *Melilotus albus* (0.41), *Trifolium campestre* (0.40), *Melilotus officinalis* (0.40), *Matricaria perforata* (0.40), *Conium maculatum* (0.39), *Bromus tectorum* (0.38), *Silene latifolia alba* (0.38), *Dipsacus fullonum* (0.36), *Calamagrostis epigejos* (0.36), *Sinapis arvensis* (0.35), *Sanguisorba minor* (0.35), *Abution theophrasti* (0.34), *Echium vulgare* (0.34), *Euphorbia seguieriana* (0.34), *Descurainia sophia* (0.33), *Lepidium ruderale* (0.32), *Anchusa officinalis* (0.32), *Carduus acanthoides* (0.32), *Torilis arvensis* (0.31), *Verbascum phlomoides* (0.31), *Artemisia vulgaris* (0.31), *Eryngium campestre* (0.30), *Avena sativa* (0.30), *Coronilla varia* (0.30), *Polygonum persicaria* (0.30)
Mid-successional site	***Prunus spinosa* (0.70), *Hypericum perforatum* (0.65), *Cornus sanguinea* (0.59), *Dipsacus laciniatus* (0.57), *Rumex patientia* (0.52), *Calamagrostis epigejos* (0.52), *Agrimonia eupatoria* (0.52), *Dasypyrum villosum* (0.51)**, *Euphorbia cyparissias* (0.49), *Lathyrus tuberosus* (0.49), *Rumex crispus* (0.49), *Rubus ulmifolius* (0.49), *Linaria vulgaris* (0.49), *Galium verum* (0.49), *Verbascum nigrum* (0.48), *Prunus persica* (0.47), *Vicia cracca* (0.47), *Senecio erucifolius* (0.46), *Clinopodium vulgare* (0.46), *Coronilla varia* (0.46), *Melilotus albus* (0.45), *Cephalaria transsylvanica* (0.42), *Stachys palustris* (0.42), *Petrorhagia prolifera* (0.42), *Tanacetum vulgare* (0.41), *Salix alba* (0.41), *Dipsacus fullonum* (0.41), *Melilotus officinalis* (0.40), *Sambucus ebulus* (0.38), *Poa trivialis* (0.38), *Centaurea stoebe* (0.38), *Carduus acanthoides* (0.38), *Odontites vernus* (0.38), *Verbascum phlomoides* (0.38), *Populus alba* (0.38), *Juglans regia* (0.37), *Tragopogon dubius* (0.37), *Silene vulgaris* (0.37), *Rosa canina* (0.36), *Equisetum arvense* (0.36), *Conium maculatum* (0.36), *Ulmus* sp. (0.35), *Silene latifolia alba* (0.35), *Populus nigra* (0.34), *Euphorbia esula* (0.34), *Epilobium hirsutum* (0.34), *Echium vulgare* (0.34), *Glycyrrhiza echinata* (0.34), *Bromus arvensis* (0.33), *Xeranthemum cylindraceum* (0.33), *Scabiosa ochroleuca* (0.33), *Medicago falcata* (0.33), *Malus domestica* (0.33), *Centaurea arenaria* (0.33), *Avena fatua* auct. (0.32), *Prunus cerasifera* (0.32), *Triticum aestivum* (0.32), *Crepis biennis* (0.32), *Cirsium vulgare* (0.32), *Torilis arvensis* (0.31), *Artemisia vulgaris* (0.31), *Rumex conglomeratus* (0.30), *Picris hieracioides* (0.30), *Holcus lanatus* (0.30), *Cruciata laevipes* (0.30), *Bromus hordeaceus* (0.30), *Epilobium tetragonum* (0.30)
Park	***Quercus robur*** (0.51), *Philadelphus coronarius* (0.45), *Viola odorata* (0.33), *Symphoricarpos albus* (0.31), *Spiraea media* (0.31)
Residential area-compact	***Digitaria ciliaris* (0.71), *Campsis radicans* (0.63), *Syringa vulgaris* (0.51), *Kerria japonica* (0.50)**, *Ligustrum vulgare* (0.47), *Armoracia rusticana* (0.47), *Vitis vinifera* (0.46), *Antirrhinum majus* (0.44), *Ficus carica* (0.43), *Aquilegia* sp. (0.43), *Mirabilis jalapa* (0.41), *Rhus typhina* (0.41), *Hibiscus syriacus* (0.40), *Lonicera japonica* (0.39), *Ipomoea purpurea* (0.39), *Albizia julibrissin* (0.38), *Vinca major* (0.38), *Rudbeckia hirta* (0.38), *Fragaria vesca* (0.36), *Oenothera biennis* (0.36), *Iris germanica* (0.35), *Coreopsis tinctoria* (0.35), *Prunus domestica* (0.34), *Bassia scoparia* (0.34), *Viola odorata* (0.33), *Anethum graveolens* (0.33), *Lactuca saligna* (0.33), *Geranium pusillum* (0.33), *Thuja orientalis* (0.33), *Cosmos bipinnatus* (0.32), *Tagetes patula* (0.32), *Crepis biennis* (0.32), *Alcea rosea* (0.32), *Ranunculus repens* (0.32), *Hedera helix* (0.32), *Oxalis corniculata* (0.31), *Mentha piperita* agg. (0.31), *Impatiens balfourii* (0.30), *Veronica arvensis* (0.30)
Residential area-open	***Mirabilis jalapa* (0.50)**, *Spiraea media* (0.39), *Duchesnea indica* (0.38), *Solanum tuberosum* (0.36), *Acer pseudoplatanus* (0.36), *Ajuga reptans* (0.35), *Sedum kamtschaticum* (0.35), *Commelina communis* (0.34), *Vinca major* (0.34), *Ligustrum vulgare* (0.33), *Rumex pulcher* (0.33), *Viola odorata* (0.33), *Syringa vulgaris* (0.33), *Hibiscus syriacus* (0.33), *Tagetes patula* (0.32), *Sedum rupestre* (0.32), *Geum urbanum* (0.32), *Chelidonium majus* (0.31), *Sisymbrium officinale* (0.30), *Prunella vulgaris* (0.30), *Petunia* x *atkinsiana* (0.30), *Anagallis arvensis* (0.30)
Square	-

Taxa are listed by decreasing values of Φ (in parenthesis). Only taxa with Φ > 0.3 are shown. The taxa with Φ > 0.5 are marked bold.

**Table 2:** Characterization of the studied cities in Serbia.

City (City Code)	N (°)	E (°)	Alt (m a.s.l.)	T (°C)	ΔT (°C)	P (mm)	Climate Type/Subtype
Niš (NI)	43.32083	21.89528	199	11.36	31.66	625	transitional submediterranean-Aegean subcontinental (IV6)
Vranje (VR)	42.55472	21.89778	481	9.80	30.65	625	
Beograd (BG)	44.81583	20.46000	113	11.9	29.74	672	transitional subcontinental-semiarid continental (VI3b/VII)
Novi Sad (NS)	45.25500	19.84528	85	11.34	30.67	611	
Pančevo (PA)	44.87083	20.64083	81	11.74	29.78	644	
Šabac (ŠA)	44.75694	19.69444	81	11.56	30.67	692	
Smederevo (SD)	44.66500	20.92694	78	11.36	29.99	650	
Sremska Mitrovica (SM)	44.96806	19.60694	84	11.52	30.79	649	
Vršac (VŠ)	45.12111	21.29555	92	11.30	30.5	666	
Čačak (ČA)	43.89111	20.35000	241	10.93	30.82	785	semi-arid temperate continental (subcontinental)—central-southeastern Balkan or Moesian (IV3)
Kragujevac (KG)	44.01000	20.91667	177	11.03	31.01	690	
Kruševac (KŠ)	43.58194	21.32639	162	11.16	31.55	652	
Loznica (LO)	44.53361	19.22389	126	11.14	29.73	844	
Novi Pazar (NP)	43.14028	20.51722	495	9.97	31.28	794	
Pirot (PI)	43.15611	22.58528	371	10.26	31.83	617	
Valjevo (VA)	44.26861	19.88417	188	10.96	30.70	803	
Kikinda (KI)	45.83000	20.46500	83	11.31	31.61	557	semi-arid continental Pannonian (VII)
Sombor (SO)	45.77278	19.11500	90	11.16	30.85	602	
Subotica (SU)	46.10000	19.66500	116	10.98	31.13	555	
Zrenjanin (ZR)	45.38028	20.39083	84	11.55	31.22	572	
Negotin (NG)	44.22806	22.53056	47	11.33	32.13	603	semi-humid continental Danubian (VII)
Zaječar (ZA)	43.90333	22.27833	132	10.88	31.92	623	
Užice (UE)	43.85667	19.84028	414	9.52	29.16	899	humid temperate-continental-west Balkan or Illyrian (IV2b)
Sjenica (SJ)	43.27306	20.00028	1006	6.48	30.91	755	humid mountain alpine (XI)

N—latitude, E—longitude, Alt—elevation, T—mean annual temperature, ΔT—difference between mean temperature in July and January, P—precipitation.

**Table 3:** Total explained variation, the influence of habitat type, climate, and their shared effect on the plant species composition in the analyzed cities.

	Habitat Type	Climate	Shared Effect
Unadjusted R2 (%)	12.7	3.8	<0.1
Adjusted R2 (%)	9.7	2.3	0

## Data Availability

The data are not publicly available due to the database is part of the Ph.D. thesis. The complete database will be available only after the publication of the dissertation in 2022. Also, we will use the same database for other articles we plan to publish. For these reasons, we believe that the complete database should not be publicly available at this moment.
